# Systemic and Oral Characteristics of Convalescent Inpatients Requiring Oral-Health Management by a Dental Specialist during Hospitalization

**DOI:** 10.3390/geriatrics9030082

**Published:** 2024-06-17

**Authors:** Naoki Todayama, Ryuzo Hara, Tomohiro Tabata, Yukiko Hatanaka, Tomoko Mukai, Mika Someya, Miki Kuwazawa, Hiroyuki Suzuki, Shouji Hironaka, Nobuyuki Kawate, Junichi Furuya

**Affiliations:** 1 Department of Oral Function Management, Graduate School of Dentistry, Showa University, Tokyo 145-8515, Japan; ed20-n201@grad.showa-u.ac.jp (N.T.); r.hara@dent.showa-u.ac.jp (R.H.); gd22-t014@dent.showa-u.ac.jp (T.T.); y.hatanaka@dent.showa-u.ac.jp (Y.H.); gd23-m012@dent.showa-u.ac.jp (M.S.); miki@dent.showa-u.ac.jp (M.K.); h.suzuki@dent.showa-u.ac.jp (H.S.); 2 Division of Oral Function Management, Department of Oral Health Management, School of Dentistry, Showa University, Tokyo 145-8515, Japan; t.mukai@dent.showa-u.ac.jp; 3 Dental Department, Fujigaoka Hospital, Yokohama 227-8501, Japan; 4 Department of Hygiene and Oral Health, Graduate School of Dentistry, Showa University, Tokyo 145-8515, Japan; hironaka@dent.showa-u.ac.jp; 5 Department of Rehabilitation Medicine, Graduate School of Medicine, Showa University, Tokyo 142-8555, Japan; kawate@med.showa-u.ac.jp

**Keywords:** convalescent hospital, dentures, dysphagia, oral health, rehabilitation, malnutrition

## Abstract

Older adults often experience poor oral functions, hindering rehabilitation post-acute disease treatment. However, characteristics of hospitalized patients who would benefit from professional oral-health management (POHM) have not been clarified. Therefore, we aimed to elucidate systemic and oral characteristics of patients requiring POHM during hospitalization in a convalescent hospital. This study included 312 participants admitted to the rehabilitation department of a convalescent hospital for a year. The patients were categorized according to POHM requirements (no-POHM group: 137 patients; POHM group: 175 patients) by discharge. Age, sex, primary disease at admission, Glasgow coma scale (GCS), Functional Independence Measurement (FIM), Mini nutritional assessment-short form (MNA-SF), Functional oral intake scale (FOIS), number of present and functional teeth, Oral Health Assessment Tool (OHAT) scores, and POHM details provided during patient hospitalization were compared. Binomial logistic-regression analysis identified patients requiring POHM as those who had suffered a stroke and had a low number of present teeth, poor overall oral health, low food form, and low motor skills at admission. A high percentage of POHM interventions comprised oral-hygiene care and denture treatment. In summary, patients whose oral health has deteriorated and those experiencing oral-intake difficulties upon admission to a convalescent hospital may require oral-health management.

## 1. Introduction

The oral health status of older convalescent inpatients often declines during acute treatment and hospitalization [1]. Even with appropriate oral health management under a multidisciplinary approach, it is difficult to improve oral health in the acute phase alone [2]. Therefore, seamless oral health management from the acute to the convalescent phase is crucial as patients recover in convalescent hospitals. In particular, oral health status at admission to a convalescent hospital may be associated with activities of daily living (ADL) and nutritional status [1,3], and poor oral hygiene at admission has been associated with poor ADL at discharge [4]. Further, dry mouth and denture status are associated with eating patterns [5], and oral hygiene status is related to length of stay and home discharge rates [6]. In recent years, hospitalized patients have become increasingly older. This population experiences not only deteriorating oral hygiene but also a decline in oral function due to factors such as fewer remaining teeth and ill-fitting dentures [7], highlighting the need to focus on both oral function and oral hygiene. Thus, an integrated approach to oral health management, including oral function in addition to oral hygiene, is important in hospitalized patients.

Oral health issues extend beyond impacting systemic diseases like aspiration pneumonia [8]; they also encompass challenges related to dentures, occlusal support, and chewing ability [9], all of which can hinder food intake during hospitalization. Poor nutrition resulting from these difficulties further undermines motor function [10]. Therefore, establishing a dental department within a convalescent hospital and implementing professional oral-health management (POHM) can significantly enhance oral health for patients requiring assistance with oral function. Convalescent hospitals aim for multidisciplinary rehabilitation to facilitate patients’ recovery and eventual discharge. Given the pivotal role of nutrition in effective rehabilitation, maintaining good oral health becomes paramount for proper nutritional management [11]. Therefore, oral health care for patients admitted to convalescent hospitals is critical.

The convalescent period offers an opportune environment for implementing comprehensive oral health management due to prolonged hospital stay and stable disease status. However, in practice, such management often falls short of its potential [1]. Reasons for this shortfall may include a scarcity of dental professionals within convalescent hospitals and difficulties in conducting appropriate oral health assessments by nurses and other multidisciplinary professionals [12]. Consequently, leveraging patient characteristics upon admission to identify those likely to benefit from oral health management holds promise for enhancing its effectiveness and efficiency in convalescent settings. Thus, this study aimed to identify the characteristics of patients requiring POHM during their admission to convalescent hospitals.

## 2. Materials and Methods

### 2.1. Participants

**Figure 1:**
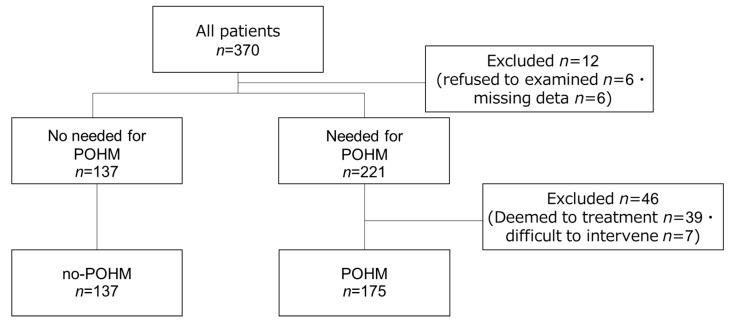
Research flow chart.

This study was conducted in Kanagawa Prefecture, Japan, and included 370 patients admitted to a convalescent hospital from August 2021 to August 2022. The study flowchart is presented in Figure 1. Six patients who refused to be examined by the dentist during hospitalization and another six patients with missing data were excluded from the study. A total of 58 patients were excluded, including 46 patients who were deemed to require POHM by a dentist at the time of admission but were unable to give informed consent. In this study, all patients admitted to the rehabilitation department of a convalescent hospital underwent an oral health assessment within approximately 1 week of admission. The inclusion and exclusion criteria are shown in Table 1. The assessment, encompassing caries, periodontal status, denture condition, and oral hygiene levels, was conducted by four pre-calibrated dentists, who had over 5 years of clinical experience, specializing in geriatric dentistry. The need for oral health management was comprehensively evaluated based on the assessment results, patient preferences, and input from various professionals, including nurses. Subsequently, patients were categorized into three levels: no need (ward nurses’ management suffices), oral health management level (requiring oral health management by DH or dentists), and oral function management level (necessitating oral function management, including dental treatment by dentists), following consultations with the four assessing dentists. Patients who gave their consent were provided with professional oral hygiene management and oral function management by dentists (POHM). Additionally, the content of POHM was investigated for patients classified at the oral hygiene management and oral function management levels. Through a retrospective study of medical records, we examined medical and conference records during hospitalization up to the time of discharge and classified the patients into two groups: those who received POHM during hospitalization (POHM group) and those who did not need it (no-POHM group, as control). We also compared their general and oral health information at admission. Informed consent was obtained from all study participants using the opt-out method before starting the study. This study was approved by the Showa University Research Ethics Review Board (Approval No. 22-002-B).

### 2.2. Outcomes

The survey items were as follows: age, sex, main disease, level of consciousness (Glasgow Coma Scale [GCS]), degree of independence (Functional Independence Measurement [FIM]), nutritional status (Mini nutritional assessment-Short Form [MNA-SF]), nutritional methods (Functional Oral Intake Scale [FOIS]), number of present teeth, number of functional teeth, Oral Health Assessment Tool (OHAT) score, need for POHM during hospitalization, and details of POHM performed during hospitalization. The “main disease” was the disease that caused the patient to be admitted to a convalescent hospital. Moreover, patients were classified according to whether they had suffered a stroke. The GCS assesses “eye opening”, “speech”, and “movement”, with higher scores indicating a better level of consciousness [13]. The FIM consists of 13 motor items and 5 cognitive items, totaling 18 items, each of which is classified into seven levels [14]. A score of 7 indicates complete independence; 6, modified independence; 5, supervision; 4, minimal assistance; 3, moderate assistance; 2, maximum assistance; and 1, full assistance. The MNA-SF scores six items (decrease in food intake over 3 months, weight loss over 3 months, gait status, presence of mental stress or acute illness, presence of neurological or psychological problems, and body mass index or calf circumference) on a scale ranging from 0 to 2 points each, with higher values indicating better nutritional status [15]. FOIS is a method for evaluating oral intake, with seven levels ranging from tube feeding to oral intake, with higher values indicating a better diet [16]. The number of present teeth was defined as the number of teeth that are partially or fully erupted into the oral cavity. The number of functional teeth included natural and prosthetic teeth and dentures and implants; teeth with more than 3 degrees of movement and remaining roots were excluded. OHAT values were extracted for comprehensive oral health status. The OHAT is an assessment tool developed by Chalmers for comprehensive oral assessment of institutionalized older residents in need of care [17]. The OHAT is divided into eight categories (lips, tongue, gingiva/mucosa, saliva, remaining teeth, dentures, oral cleaning, and toothache) and evaluated on a three-point scale ranging from healthy (0 points) to slightly poor (1 point) and morbid (2 points). Additionally, the contents of the POHM actually performed during hospitalization were extracted in the form of multiple responses, and divided into denture treatment, caries treatment, tooth extraction, dysphagia rehabilitation, and others.

### 2.3. Statistical Analysis

Mann–Whitney U and χ^2^ tests were used to compare the groups with and without POHM by dentists during hospitalization. Binomial logistic-regression analysis was performed, with the presence or absence of professional oral-health management (POHM) by dentists during hospitalization (0: no management, 1: management) serving as the outcome variable. Age, sex, stroke status (0: presence of stroke, 1: absence of stroke), number of present teeth, OHAT total score, FOIS, FIM motor, FIM cognition, and MNA-SF total score were included as predictor variables to assess the general and oral characteristics of the group receiving POHM during hospitalization. Selection of predictor variables was based on their clinical significance and consideration of multicollinearity, with guidance from previous studies [1,2,3,4,5,11]. The model’s goodness of fit was evaluated using the Hosmer–Lemeshow goodness-of-fit test. Statistical analysis was performed using SPSS Ver.27 (IBM Tokyo, Japan), and the significance level for all statistical processing was set at 5%.

## 3. Results

### 3.1. Characteristics of Systemic and Oral Function during Convalescent Hospitalization for Inpatients Requiring Professional Oral-Health Management

Table 2 presents the basic demographic information of the study participants. Among the 312 participants, 137 (mean age 73.4 ± 13.5) did not receive POHM (no-POHM group) during hospitalization, while 175 (mean age 76.6 ± 10.7) received POHM (POHM group). The GCS total score, primary disease, presence of stroke, FIM motor, FIM cognition, MNA-SF, FOIS, and number of current teeth were evaluated. For participants without POHM, the GCS total score was 14.7 ± 1.25, whereas for those receiving POHM, it was 13.9 ± 2.4, indicating a lower level of consciousness among the latter group. FIM motor and FIM cognition scores were 56.4 ± 18.4 and 30.3 ± 7.2, respectively, for participants without POHM, and 41.3 ± 21.3 and 25.8 ± 10.7, respectively, for those with POHM, suggesting decreased activities of daily living (ADL) in the latter group. The MNA-SF score was 9.7 ± 2.2 for participants without POHM, with 13.9% classified as malnourished, compared to 8.7 ± 2.5 for those with POHM, with 29.7% classified as malnourished, indicating poorer nutrition among the latter group. FOIS scores were 6.7 ± 0.6 for participants without POHM and 5.6 ± 1.9 for those with POHM, indicating decreased swallowing function in the latter group. Regarding the oral health, the number of present teeth was 16.5 ± 1.9 for participants without POHM and 21.2 ± 8.1 for those with POHM, indicating more missing teeth among the latter group. The number of functional teeth was 24.2 ± 7.3 for participants with POHM and 25.1 ± 5.3 for those without POHM, suggesting fewer functional teeth among the former group despite a higher number of present teeth. The OHAT total score was 3.6 ± 2.9 for participants with POHM, indicating poorer oral health status in this group. However, there were no significant differences in age and sex between the two groups. Based on the results of binomial logistic-regression analysis, the presence of stroke, current number of teeth, OHAT total score, FOIS, and FIM motor were identified as significant explanatory variables associated with POHM during hospitalization (Table 3). The fit of this binomial logistic-regression model was deemed satisfactory according to the result of the Hosmer–Lemeshow goodness-of-fit test (*p* = 0.119).

### 3.2. Contents of POHM

**Figure 2:**
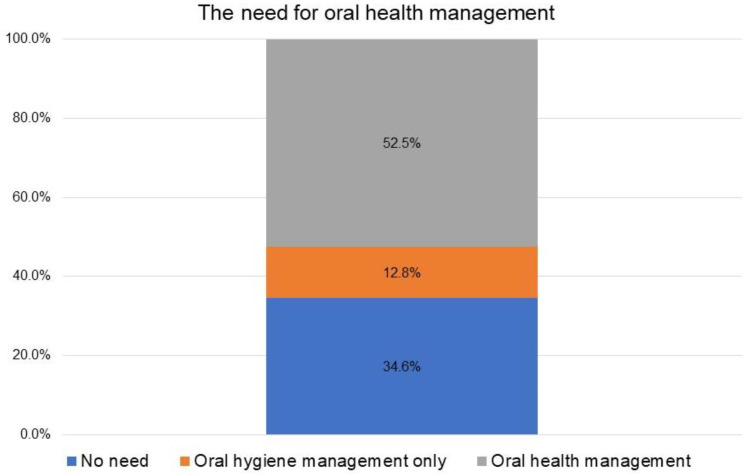
Oral function management level.

**Figure 3:**
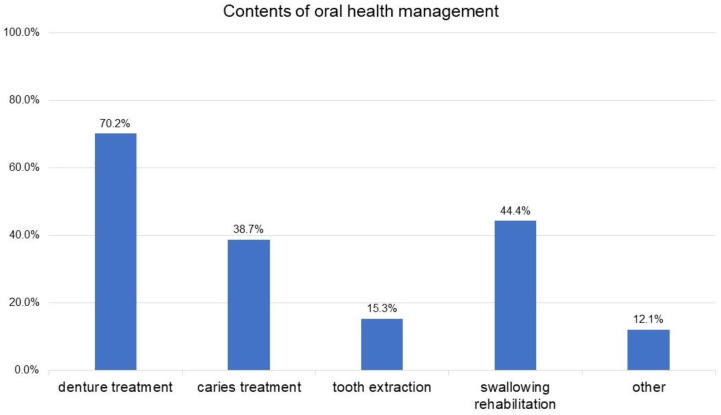
Professional oral-health management methods conducted during the hospitalization period.

Regarding the need for POHM by dentists at admission, 34.6% of patients were found to have no need, 12.8% to require oral hygiene management, and 52.5% to require oral function management (Figure 2). Denture treatment was the most common type of oral health management performed during the hospitalization period, followed by swallowing rehabilitation, caries treatment, and tooth extraction (Figure 3).

## 4. Discussion

In this study, the characteristics of patients requiring oral function management were identified among all inpatients in the rehabilitation department of a convalescent hospital. The findings underscore the necessity not only for routine oral care but also for specialized oral health management by dental professionals, particularly in areas such as denture treatment and swallowing rehabilitation. However, the availability of dental staff within hospitals is often limited, necessitating targeted approaches to patients requiring oral function management.

Furthermore, there is a pressing need for comprehensive oral health education, expansion of hospital dentistry, and improved collaboration between medical and dental teams to prevent the deterioration of oral health states. Oral health management plays an important role in rehabilitation medicine, and it is believed that multidisciplinary involvement in oral health is required to maximize the outcomes of rehabilitation, especially in convalescent hospitals with few dental professionals. Currently, however, there is a lack of medical–dental coordination and insufficient integration of oral health knowledge into rehabilitation and general medical practices. In fact, medical staff often prioritizes treatment of systemic disease, sometimes overlooking oral health considerations. Previous studies have revealed that physicians and nurses exhibit less than 30% proficiency in identifying caries and oral pathology, with dental consultation requests occurring only 32% of the time [18]. Hence, we posit that through collaborative efforts between medicine and dentistry, early detection of oral issues can be optimized. This collaborative approach involves understanding both oral and systemic characteristics necessitating oral function management, and screening for relevant factors accordingly.

The key findings of the present study are as follows. The characteristics of patients requiring professional oral health management by a dentist during convalescent hospitalization were identified as stroke, low number of present teeth, high OHAT total score indicating declined comprehensive oral health, low FOIS implying low food form, and low FIM motor score. Moreover, patients admitted to convalescent hospitals were found to have a greater need for oral function as well as oral hygiene management, and in particular a greater need for denture treatment. Notably, stroke incidence, low number of current teeth, worse overall oral health status, low nutritional methods, and low level of independence were extracted as characteristics of the POHM group, even after adjusting for confounding factors. The results were also consistent with those of a simple two-group comparison and revealed the general and oral characteristics of patients requiring POHM by a dentist during hospitalization during the convalescent period. In clinical practice, requests for dental consultations often revolve around oral function management, typically stemming from issues related to eating difficulties. However, the decline in oral function is not solely a dental concern; it is influenced by a variety of factors, including low nutrition and low activity levels, which can decline with age and systemic disease. Some dietary challenges cannot be addressed by dentistry alone. To enhance the effectiveness and efficiency of medical–dental collaboration, this study identifies characteristics of patients likely to require dental treatment. This guidance may assist hospital dentistry, particularly in settings with limited staff, in efficiently managing the oral health of inpatients who require POHM.

The categorization of participants into the POHM group and no-POHM group based on comprehensively oral evaluations by dentists may have contributed to the poorer oral health status observed in the POHM group. Nonetheless, it is important to acknowledge that participants’ systemic conditions could also impact their oral health status. Patients hospitalized in the convalescent stage have complex oral health due to aging and multiple diseases, and their oral health often deteriorates [1]. In particular, patients who have experienced stroke often present with pre-existing poor oral health. Moreover, patients with periodontal disease, one of the causes of tooth loss, may have an increased risk of having a stroke [19]. The higher OHAT score in the POHM group observed in the present study may be attributed to the inclusion of a higher number of patients who had experienced stroke. Although these are likely to receive oral health care in acute care hospitals by a multidisciplinary team, some oral health concerns, such as dentures and oral hygiene, are difficult to improve during the acute phase of treatment and recovery [2]. Notably, denture therapy was the most commonly recognized oral functional management performed in the present study. Further, appropriate denture placement can facilitate food-bolus transfer and pharyngeal swallowing function [20]. Additionally, it is important for oral health management during recovery, as it can contribute to improved feeding and swallowing function. Poor oral health at the time of recovery is associated with low independence, food form, poor nutritional status, and a low number of present teeth, regardless of the main disease [21]. Acute hospital stays are shorter than those in convalescent hospitals, and denture treatment, especially for missing teeth, is often more difficult as it requires specialized treatment by a dentist. Consequently, patients are likely to be admitted to convalescent hospitals without fully recovering from their oral health conditions.

Proper denture fit aids in food mass transfer and pharyngeal swallowing function during ingestion of foods requiring mastication [20]. In the present study, the POHM group required more denture treatment and exhibited a trend towards lower FOIS, which may have resulted in lower food form due to denture incompatibility. A decrease in the number of teeth present, especially loss of occlusal support in the molars, is associated with dysphagia, which leads to malnutrition [22]. Moreover, overall oral health is related to food form in patients with stroke [1]. The lower level of awareness and FIM perception in the POHM group may have led to complications in the preceding phase of the 5-stage model of dysphagia, potentially contributing to the lower FOIS in the group requiring POHM. Additionally, low FIM movement was extracted as a systemic characteristic of the POHM group, suggesting that motor and consciousness disorders may hinder self-care of oral cleaning and use of dentures, resulting in poor oral health status. The conditions for high ADL at the time of admission to a convalescent hospital are related to non-stroke, high nutritional status, good swallowing function, and good oral health [21]. Moreover, patients with good oral health had a higher recovery in ADL [23].

In the present study, the need for professional oral-health management by a dentist at the time of admission was 34.6% for no need, 12.8% for the level of oral hygiene management only, and 52.5% for the level of oral function management. In convalescent hospitals, the level of independence is often relatively well maintained, and patients themselves and the nurses in charge are the primary providers of daily oral health care. However, in this study, many patients required oral hygiene management by a dentist or dental hygienist, and approximately half of the hospitalized patients required oral function management. The actual composition of oral function management before discharge from the hospital was significantly higher for denture treatment, followed by swallowing rehabilitation, dental caries treatment, and tooth extraction. Previous studies have also shown that denture maladjustment and dysphagia affect food form and nutritional status [3,20,22]. Moreover, improving food form is often a priority for discharge from the hospital, and convalescent settings are conducive to intensive denture therapy and swallowing rehabilitation owing to the relatively long hospital stay, which can easily contribute to improved food form. Another possible reason for the high rates of denture treatment and dysphagia rehabilitation was that all procedures in this study were performed at the bedside or in other visiting settings, rather than in the clinic room.

The findings of this shed light on the essential characteristics of patients’ overall health and oral function that warrant focused attention for efficiently and effectively managing oral function in convalescent hospitals with limited dental human resources. In such settings, oral health management is a collaborative effort between medical and dental professionals. However, it is crucial to recognize that only dentists can provide specialized oral function management, particularly for denture-related issues. This study underscores the high treatment needs for denture treatment and swallowing rehabilitation among inpatients in convalescent hospitals, emphasizing the indispensable role of dentists in interdisciplinary healthcare teams and the imperative for comprehensive oral function management, encompassing dentures and swallowing problems.

Furthermore, the study highlights the importance of dental professionals providing oral care instructions and oral hygiene management to both patients and nurses, who primarily oversee daily oral care routines. Some of the key factors identified in this study as determinants for professional oral-health management (POHM) can be assessed using video images, facilitating their integration into tele-dental programs recognized for their utility [24]. This approach not only supports effective POHM in convalescent hospitals with limited dental personnel but also contributes to enhancing overall rehabilitation effectiveness in convalescent settings with sparse dental professional involvement.

However, the study had limitations. First, we did not examine the changes in general health and oral function due to oral health management during hospitalization, and the effects of oral health management during recovery need to be clarified in future studies. Additionally, the general condition of patients varies greatly depending on their main illness, severity of illness, and comorbidities. However, as this study only accounted for the presence or absence of stroke, the most common illness, and level of consciousness, additional measures, such as evaluation of the Charlson comorbidity index, which was proposed by Charlson et al. [25] in 1987, are necessary. Further, the longer the hospital stay, the poorer the oral health becomes [26]; however, we did not consider the number of days of hospitalization in our study. Our analysis focused solely on the components of oral function management during visits to convalescent hospitals. Furthermore, this study was conducted at a single center, and the generalizability of the results to other centers and different underlying diseases may be limited. Additionally, the small sample size made it challenging to analyze factors related to the need for POHM for each primary disease. Hence, future multicenter collaborative studies are necessary to explore disease-specific issues related to the need for POHM. Moreover, it is important to note that in the hospital under study, nurses receive comprehensive oral health education and provide active daily oral care, potentially resulting in relatively good oral health among patients. Additionally, oral function management procedures were conducted at the bedside or during other visits rather than in dedicated clinic rooms. The absence of air turbines in the bedside environment likely contributed to the low rate of dental caries procedures. Finally, selection bias may have influenced the results, as 39 patients declined dental treatment despite its necessity, and 7 patients were deemed difficult to treat due to deteriorating general conditions, potentially affecting the number of patients originally included in the POHM group.

## 5. Conclusions

This longitudinal study highlights the potential necessity of specialized oral health management by dentists for all inpatients admitted to rehabilitation departments of convalescent hospitals. Particularly, stroke patients experiencing a decline in overall health and deteriorating eating patterns upon admission may benefit from specialized oral-health management, encompassing oral hygiene management, denture treatment, and swallowing rehabilitation.

## Figures and Tables

**Table 1:** Inclusion and exclusion criteria.

Selection Criteria	Exclusion Criteria
Patients who agreed to dental examination	Patients with missing data
Patients who received a dental examination within approximately 1 week of hospitalization	Patients who refused dental examination
	Patients who needed but did not wish to receive dental treatment
	Patients who had difficulties with dental treatment

**Table 2:** Characteristics of the participants included in this study.

Characteristics	No-POHM Group	POHM Group	
	*n* = 137	*n* = 175	
	Mean ± SD/*n*(%)		*p*-Value
Age ^a^	73.4 ± 13.5	76.6 ± 10.7	0.071
Sex ^b^			
Male	69 (50.4)	85 (48.6)	0.820
Female	68 (49.6)	90 (51.4)	
GCS total score ^a^	14.7 ± 1.25	13.9 ± 2.4	<0.001 **
Primary disease ^b^			
Cerebral infarction	12 (8.8)	39 (22.3)	<0.001 **
Cerebral hemorrhage	8 (5.8)	15 (8.6)	
Subarachnoid hemorrhage	8 (5.8)	16 (9.1)	
Fracture	55 (40.1)	29 (16.6)	
Disuse syndrome	13 (9.5)	21 (12.0)	
Malnutrition	1 (0.7)	0 (0)	
Parkinson’s disease	7 (5.1)	3 (1.7)	
Other	33 (24.1)	51 (29.1)	
Stroke status ^b^			
Stroke	28 (20.4)	70 (40.0)	<0.001 **
Other	109 (79.6)	105 (60.0)	
FIM motor ^a^	56.4 ± 18.4	41.3 ± 21.3	<0.001 **
FIM cognition ^a^	30.3 ± 7.2	25.8 ± 10.7	<0.001 **
MNASF ^b^	9.7 ± 2.2	8.7 ± 2.5	0.002 *
Malnourished	19 (13.9)	52 (29.7)	<0.001 **
At risk of malnutrition	85 (62.0)	98 (56.0)	
Normal nutritional status	33 (24.1)	25 (14.3)	
FOIS ^a^	6.7 ± 0.6	5.6 ± 1.9	<0.001 **
Number of present teeth ^a^	21.2 ± 8.1	16.5 ± 1.9	<0.001 **
Number of functional teeth ^a^	25.1 ± 5.3	24.2 ± 7.3	<0.001 **
OHAT total score ^a^	1.8 ± 2.1	3.6 ± 2.9	<0.001 **

* p < 0.050, ** *p* < 0.001 ^a^ Mann–Whitney U test ^b^ χ^2^ test; FIM, Functional Independence Measurement; FOIS, Functional Oral Intake Scale; GCS, Glasgow Coma Scale; MNASF, Mini nutritional assessment-Short Form; OHAT, Oral Health Assessment Tool.

**Table 3:** Binomial logistic-regression analysis: factors related to oral-health management during convalescent hospitalization.

	B	Standard Error	Wald	Exp (B)	*p*-Value	95% CI
Age	0.009	0.013	0.487	1.009	0.485	0.984–1.035
Sex	0.172	0.289	0.353	1.187	0.552	0.674–2.090
Stroke status	0.653	0.329	3.943	1.922	0.047 *	1.009–3.663
Number of present teeth	−0.042	0.015	7.592	0.959	0.006 *	0.930–0.988
OHAT total score	0.207	0.063	10.884	1.229	<0.001 **	1.087–1.390
FOIS	−0.469	0.163	8.294	0.626	0.004 *	0.455–0.861
FIM motor	−0.024	0.009	7.279	0.976	0.007 *	0.960–0.993
FIM cognition	0.016	0.021	0.571	1.016	0.450	0.975–1.058
MNASF total score	0.012	0.061	0.037	1.012	0.847	0.897–1.141

* *p* < 0.05, ** *p* < 0.001; Objective variable: presence of professional oral-health management during hospitalization (0: no management, 1: management); FOIS, Functional Oral Intake Scale; FIM, Functional Independence Measurement; OHAT, Oral Health Assessment Tool; MNASF, Mini nutritional assessment-Short Form; CI, confidence interval. Sex (0 = man, 1 = woman), Stroke (0 = not Stroke, 1 = Stroke), OHAT total (0 = healthy, 16 = morbid), FOIS (1 = nothing by mouth, 7 = total oral diet with no restrictions).

## Data Availability

The datasets generated or analyzed during this study are available from the corresponding author upon reasonable request.
